# Multimodal Approach in Dry Eye Disease Combining In Vivo Confocal Microscopy and HLA-DR Expression

**DOI:** 10.1167/tvst.13.8.39

**Published:** 2024-08-23

**Authors:** Benjamin Blautain, Ghislaine Rabut, Bénédicte Dupas, Luisa Riancho, Hong Liang, Jade Luzu, Antoine Labbé, Jean-Sébastien Garrigue, Françoise Brignole-Baudouin, Christophe Baudouin, Karima Kessal

**Affiliations:** 1 Hôpital National de la Vision des 15-20, INSERM-DGOS CIC1423, IHU FOReSight, Paris, France; 2 Hôpital National de la Vision des 15-20, Service 3, Paris, France; 3 Sorbonne Université UM80, INSERM UMR 968, CNRS UMR 7210, Institut de la Vision, IHU ForeSight, Paris, France; 4 Ambroise Paré, APHP, Service d'Ophtalmologie, Université Paris Saclay, Boulogne, France; 5 SANTEN SAS, Ophthalmic Innovation Center, Evry-Courcouronnes, France; 6 Hôpital National de la Vision des 15-20, Laboratoire d'Ophtalmobiologie, Paris, France; 7 Université Paris Cité, Faculté de Pharmacie, Paris, France

**Keywords:** dry eye disease (DED), in vivo confocal microscopy (IVCM), grading score, HLA-DR, ocular surface

## Abstract

**Purpose:**

The purpose of this study was to determine the association between corneal images provided by in vivo confocal microscopy (IVCM) with clinical parameters and conjunctival expression of HLA-DR antigen in patients with dry eye disease (DED).

**Methods:**

Two hundred fourteen eyes of 214 patients with DED were analyzed, consisting of 2 groups of patients – 63 with autoimmune dry eye disease (AIDED) and 151 with non-autoimmune dry eye disease (NAIDED). Patients underwent a full clinical examination, including symptom screening, using the Ocular Surface Disease Index (OSDI) questionnaire, and objective analysis of DED signs by Schirmer's testing, tear break-up time (TBUT), Oxford's test, and IVCM corneal imaging. The IVCM scoring criteria were based on corneal sub-basal nerve density (ND), nerve morphology (NM), and inflammatory cell (IC) density. Quantification of conjunctival HLA-DR antigen was performed by flow cytometry.

**Results:**

The total IVCM score (T-IVCM) as well as the IVCM-IC subscore (sc) were positively correlated with HLA-DR levels with *r* = 0.3, *P* < 0.001 and *r* = 0.3, *P* < 0.01, respectively in the total population of patients with DED. The IVCM-NDsc was negatively correlated with TBUT in patients with AIDED (*r* = −0.2, *P* < 0.05) and with the Schirmer's test in patients with NAIDED (*r* = −0.24, *P* < 0.05). However, the IVCM-NMsc was positively correlated with the Oxford score only in patients with AIDED (*r* = 0.3, *P* < 0.05).

**Conclusions:**

The proposed IVCM scoring system showed significant correlations with clinical parameters along with conjunctival HLA-DR quantification in patients with DED.

**Translational Relevance:**

The IVCM grading score represents an interesting point of commonality among clinical parameters, imaging, and molecular investigation of the ocular surface.

## Introduction

Dry eye disease (DED) is a complex, significant health concern and a frequently disabling disease, the definition of which has recently been refined by the Tear Film and Ocular Surface Society Dry Eye Workshop (TFOS DEWS) II expert group,^1^ as a “multifactorial disease of the ocular surface characterized by a loss of homeostasis of the tear film, and accompanied by ocular symptoms, in which tear film instability and hyperosmolarity, ocular surface inflammation and damage, and neurosensory abnormalities play etiological roles.” Its diagnosis, often challenging, and its causes, varied and frequently intertwined, make DED a disease in its own right within ophthalmology. Diagnosis of DED requires meticulous clinical examination, including symptom screening via the use of specific questionnaires, such as the Ocular Surface Disease Index (OSDI),^2^ corneal and conjunctival staining for the Oxford score,^3^ and assessment of the quantity and the quality of tear production via the Schirmer's test and tear break-up time (TBUT), respectively.^4^^,^^5^ However, the lack of a gold standard symptom or clinical sign, along with the insufficiently understood discordance^6^ between symptoms and signs, make DED evaluation and stratification a major challenge for clinicians and researchers. This issue has contributed to a growing interest in developing multimodal methods of evaluating DED, in particular, imaging^7^^,^^8^ and biological markers, including proteomic,^9^^–^^11^ transcriptomic,^12^^,^^13^ and lipidomic^14^^,^^15^ approaches.

Among imaging techniques, in vivo confocal microscopy (IVCM) allows a morphological, minimally invasive, high-resolution, real-time evaluation of the ocular surface, including the cornea.^16^ In DED, the corneal sensory nerves and inflammatory cells, located at the level of the sub-Bowman layer, represent two parameters of interest, widely described using IVCM imaging.^17^^–^^19^ Morphological parameters of corneal nerves fibers, such as reflectivity, tortuosity, and density, allow qualitative and quantitative assessment of the cornea by IVCM imaging.^19^ Indeed, several studies demonstrate the correlation between the alteration of corneal sensory nerves and disease severity.^20^^–^^25^ Additionally, corneal IVCM images allow visualization of immune and inflammatory cells, mainly dendritic cells (DCs). Indeed, the DCs are known to play a role in regulating corneal homeostasis and responding to foreign antigens, including infectious agents.^26^ In patients with DED, the density of DCs, as well as their morphological patterns, have been correlated with disease severity.^27^^–^^30^ Interestingly, several studies suggested a close interaction between corneal sensory nerves and DCs in both healthy patients and patients with DED.^19^^,^^31^^–^^33^ This intimate relationship between DCs and corneal nerves has also been reported in an experimental DED mouse model,^34^ suggesting a role of corneal nerves in mediating immune responses. Unfortunately, due to a lack of consensus in the interpretation of IVCM images differentiating physiological from pathological parameters remains a point of debate.^35^ However, the use of imaging scores based on changes in the corneal parameters described above might facilitate IVCM evaluation, and, therefore, patient management. Recently, IVCM scores have been described in ocular surface diseases, including Meibomian gland dysfunction (MGD)^36^ and nephropathic cystinosis.^37^ Nevertheless, no consensual IVCM score taking into account the DCs and nerves has yet been described for DED. Moreover, in order to refine the evaluation of patients with DED through morphological assessment, molecular identification of the ocular surface might lead to a better description of cellular events. Indeed, considering the complexity of a DED diagnosis, several studies have been conducted in search of reliable biological markers correlated with pathophysiological disease patterns. During the past decade, the assessment of human leukocyte antigen-DR (HLA-DR) expression in conjunctival cells, has demonstrated usefulness in clinical trials reflecting levels of ocular surface inflammation.^38^^,^^39^ Conjunctival expression of HLA-DR has been correlated with clinical symptoms and signs^39^ and has been used for monitoring the effects of topical anti-inflammatory drugs in patients with DED.^40^ Therefore, based on changes in HLA-DR expression in patients with DED and its evaluation in several multicenter clinical trials, HLA-DR has been found to be a potential biomarker of DED severity and prognosis. Thus, in order to better understand the ocular surface dysregulation occurring in patients with DED, we aimed to describe a more specific profile of patients with DED via IVCM corneal images and to assess the interplay between clinical data and conjunctival HLA-DR expression with IVCM stratification. Herein, we propose a simplified IVCM score, which we found to be correlated with clinical parameters and HLA-DR molecular assessment of the ocular surface in a large population of patients with DED.

## Materials and Methods

### Study Design

This retrospective study was conducted at the “Centre Hospitalier National d'Ophtalmologie des Quinze-Vingts,” Paris, France. All patients provided written informed consent. The described research methods and analyses adhered to the tenets of the 1964 Declaration of Helsinki, and the Committee for Protection of Persons (CPP Ile-de-France A02800-55) has provided a favorable approval to this study.

### Participants

The study population (Table 1) included 214 eyes of 214 patients with DED – 63 patients with auto immune dry eye disease (AIDED) and 151 with non-auto immune dry eye disease (NAIDED). Inclusion criteria consisted of a minimum age of 18 years and a comprehensive examination at the Clinical Investigation Center specialized in DED. The diagnosis was adapted through the clinical information attested by the clinician, according to the biological indication and previous care visits. The classification of a patient with DED was tailored by the TFOS DEWS II Definition and Classification Report and Diagnostic Methodology report.^1^^,^^41^ The diagnosis of autoimmune context was made by the referring internist, in accordance with the criteria defined by American College of Rheumatology (ACR) and European League Against Rheumatism (EULAR).^42^ All of the referred patients received a variety of dedicated medical care to prevent DED, such as artificial tears, topical cyclosporine, topical antibiotics, punctal plugs, eyelid hygiene, and/or topical anti-allergic treatment. Additionally, the included population correspond to a heterogeneous population to capture a broad spectrum of patients with DED. Briefly, the clinical examination included the OSDI, Schirmer's test, TBUT, and Oxford score. Clinical assessment of symptoms and signs related to DED was followed by acquisition of IVCM images of the corneal sub-basal plexus. The worst eye of each patient was selected, based on the Oxford score. If both eyes had similar Oxford scores, the most painful eye was selected. In case of equal discomfort or pain in both eyes, a randomization between the two eyes was performed.

**Table 1.** Demographic and Dry Eye Parameters

Patients	All DED (*n* = 214)	AIDED (*n* = 63)	NAIDED (*n* = 151)	*P* Value
Female/Male	77%/23%	97%/3%	68%/32%	<0.0001***
**Mean ± SD**				
Age, y	55 ± 15	57 ± 12	55 ± 15	ns
OSDI-1 ocular symptoms	12 ± 4.6	13 ± 3.6	11 ± 5	0.0015**
OSDI-2 vision-related function	7.9 ± 4.6	9 ± 4.3	7.4 ± 4.8	0.0381*
OSDI-3 environmental triggers	7.7 ± 3.9	8.4 ± 3.8	7.3 ± 3.9	0.0532
OSDI total score	60 ± 23	67 ± 19	56 ± 24	0.0040*
Oxford score	1.1 ± 1.3	1.7 ± 1.5	0.8 ± 1.1	<0.0001****
Schirmer's tear test	15 ± 11	9.7 ± 9.8	17 ± 10	<0.0001****
TBUT	5.7 ± 3.6	4.2 ± 2.7	6.4 ± 3.7	<0.0001**
1-IVCM-[IC] subscore	1.7 ± 0.7	1.7 ± 0.6	1.7 ± 0.7	ns
2-IVCM-[ND] subscore	0.4 ± 0.5	0.4 ± 0.5	0.4 ± 0.5	ns
3-IVCM-[NM] subscore	1.5 ± 0.6	1.4 ± 0.6	1.5 ± 0.6	ns
4-IVCM total score	3.6 ± 1.1	3.5 ± 1	3.6 ± 1.1	ns
HLA-DR expression [abc]	70,000 ± 65,000	72,000 ± 62,000	70,000 ± 66,000	ns

AIDED, autoimmune dry eye disease; HLA-DR, human leukocyte antigen-DR; IVCM, in vivo confocal microscopy; NAIDED, non-autoimmune dry eye disease; ns, not significant; OSDI, Ocular Surface Disease Index; SD, standard deviation; TBUT, tear break-up time.

Patients’ clinical and HLA-DR information are presented, as well as the comparison between the DED groups, AIDED and NAIDED. Values are presented as means and standard deviations. Comparisons between mean values of each parameter in DED groups (AIDED and NAIDED) were performed using a nonparametric test with a significant *P* value presented as:

*P < 0.05; **P < 0.01; ***P < 0.001.

### Acquisition and Scoring of IVCM Images

**Figure 1.**
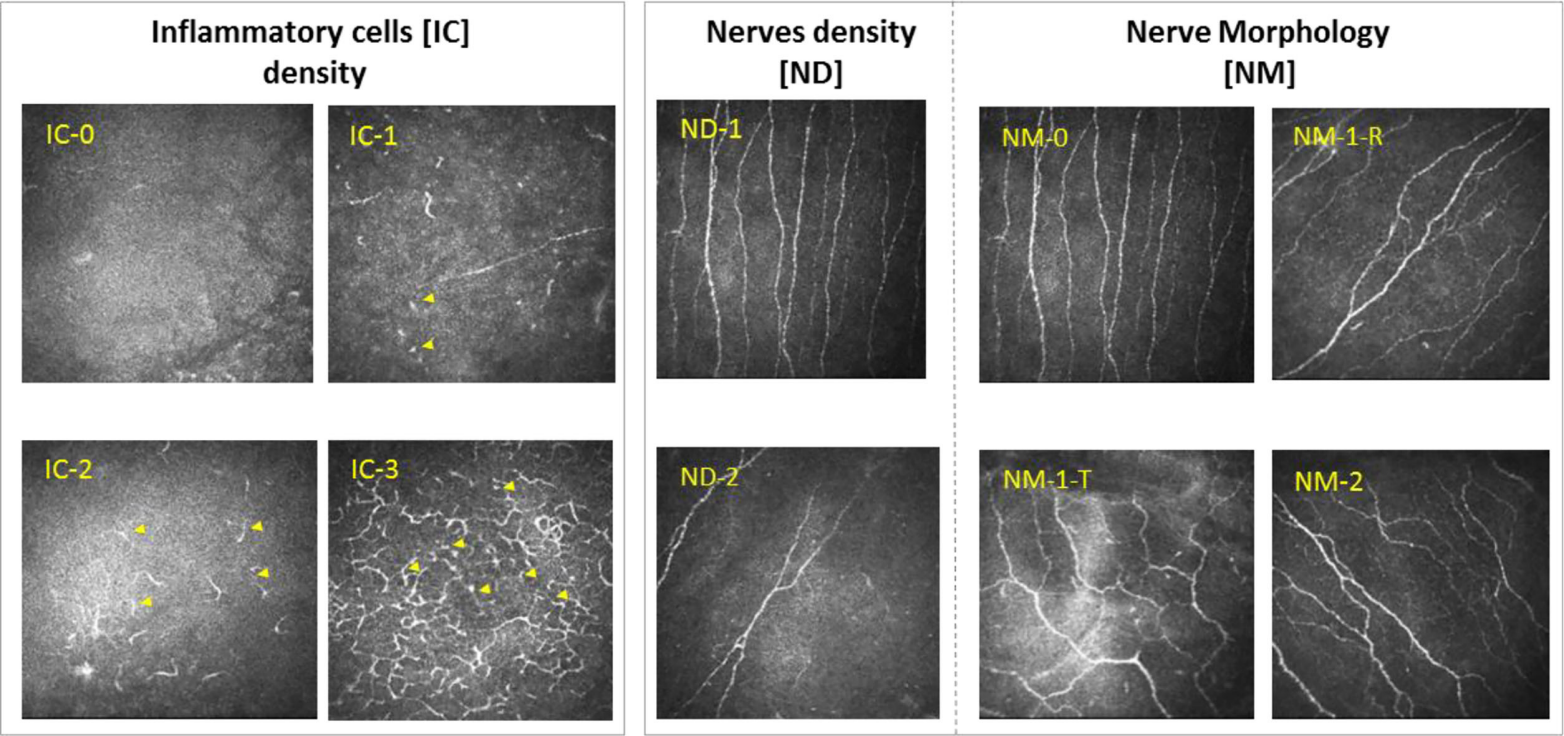
Representative images of in vivo confocal microscopy at the level of the corneal sub-basal nerve plexus in patients with DED, along with the IVCM grading score. Cellular and sensory nerve morphometric criteria were used to score images. Dendritic cells (DCs) are indicated with *yellow arrows* highlighting the increase in IC density from scores of IC-0 to IC-3. Nerve alterations are considered according to their density and their morphological patterns, reflectivity, tortuosity, or both. ICs, inflammatory cells; ND, nerve density; NM, nerve morphology; NM1-T, tortuosity; NM1-R, reflectivity; NM-2, both reflectivity and tortuosity.

Following topical anesthesia (oxybuprocaine hydrochloride 0.4%; Théa Pharma, France), the central four millimeters of the patients’ corneas were analyzed at the level of the sub-Bowman layer, where the sub-basal nerve plexus and inflammatory cells are located. The Heidelberg Retina Tomograph II combined with the Rostock Cornea Module (HRT II-RCM; Heidelberg Engineering, Heidelberg, Germany) were used to obtain IVCM images covering an area of 400 × 400 µm, digitally converted into images of 384 × 384 pixels. An IVCM grading score was then constructed, based on the three following parameters: subjective evaluation of sub-basal nerve density,^9^ nerve morphology (NM), and inflammatory cell (IC) density (Table 2). All linear hyper-reflective branching structures were considered to be nerves. All observed cells, from round, presumed immature cells to well-defined, presumably mature dendriform cells, were considered to be ICs. For IC density evaluation, the five sharpest images of each eye were chosen, based on the quality of contrast and focus (ImageJ software, U. S. National Institutes of Health, Bethesda, MD, USA, https://imagej.nih.gov/ij/). ImageJ software (1997–2018) was used to count the number of cells per image, using the multipoint tool in manual mode. The average density of ICs was then calculated and classified into the 4 categories reported in Table 2. The Total IVCM score (T-IVCM) was obtained by adding the scores of each of the three IVCM subscore (sc) parameters: NDsc (range = 0–1), NMsc (range = 0–2), and ICsc (range = 0–3). The morphometric evaluations of the corneal IVCM images were carried out in a manner blinded with regard to diagnosis and clinical outcomes, and the test-retest reliability coefficient of the IVCM score was 0.88 (*P* < 0.0001). Representative corneal IVCM images of the patients are presented in Figure 1. In a second time, analysis of (1) corneal nerve fiber density (NFD) as numbers per square millimeter (n/mm^²^), (2) corneal nerve fiber length (NFL) as millimeters per square millimeter (mm/mm²) defined as the sum of the nerve branches observed within a frame, and cell inflammatory density as n/mm²) was performed manually using Neuron J. Nerve tracing was examined using Neuron J a Java-based image analysis software package that includes a nerve-tracing plugin module.

**Table 2.** IVCM Grading Score Composition


Inflammatory cells		
Cell Density (No. Of Cells/mm^2^)	Code	ICsc
Low (<10)	IC-0	**0**
Medium low (10–49)	IC-1	**1**
Medium high (50–99)	IC-2	**2**
High (>100)	IC-3	**3**
Sensory nerves		
Density description	Code	NDsc
Normal (>15 number of nerve/mm²)	ND-1	**0**
Decreased (≤15 number of nerve/mm²)	ND-2	**1**
Morphology description	Code	NMsc
Normal	NM-0	**0**
Reflectivity	NM1-R	**1**
Tortuosity	NM1-T	**1**
Reflectivity and tortuosity	NM-2	**2**

ICs, inflammatory cells; ND, nerve density; NM, nerve morphology; NM1-T, tortuosity; NM-2, both reflectivity and tortuosity; NMI-R, reflectivity; sc, subscore.

Total IVCM (T-IVCM) is obtained by adding IVCM-IC, -ND and -NM subscores. Minimum T-IVCM score values are 0; maximum are 6.

### Flow Cytometry Analysis of Conjunctival HLA-DR Expression

Conjunctival cells were collected using conjunctival imprints (EyePrim device; Opia, France) from each patient with DED, and the polyether sulfone membranes were placed on the superior or superotemporal bulbar conjunctiva. The collected membranes were then placed into a tube containing 0.05% paraformaldehyde in phosphate-buffered saline solution and processed as soon as possible. Briefly, as described previously,^38^^,^^39^ cells were extracted from the membranes then analyzed after indirect HLA-DR antigen immunostaining (DAKO, Denmark, M0746 mouse monoclonal anti-human HLA-DR antigen alpha-chain clone TAL.1B5 as the primary antibody and F0479 goat F(ab’)2 anti-mouse FITC-conjugated as the secondary antibody) using a flow cytometer (Cytomics FC500 MCL; Beckman Coulter, USA). The quantified fluorescence intensity was derived from the HLA-DR mean fluorescence intensities (MFIs) converted into arbitrary units of fluorescence (AUF) using fluorescence calibrated beads (QIFIKIT; Dako, Denmark).

### Statistical Analysis

Statistical analyses were carried out with Prism software, version 8.0.1 (GraphPad Software Inc., San Diego, USA). A Spearman's correlation was conducted to correlate the T-IVCM score and the three related subscores (ICsc, NDsc, and NMsc), as well as HLA-DR levels, with the clinical parameters. The mean values of each quantified parameter were compared using Mann-Whitney tests. Any *P* values < 0.05 were considered statistically significant for comparison and correlation and marked with an asterisk.

## Results

### Correlations of IVCM Scores With Clinical Parameters and HLA-DR Levels in the Total Population of Patient With DED 

**Figure 2.**
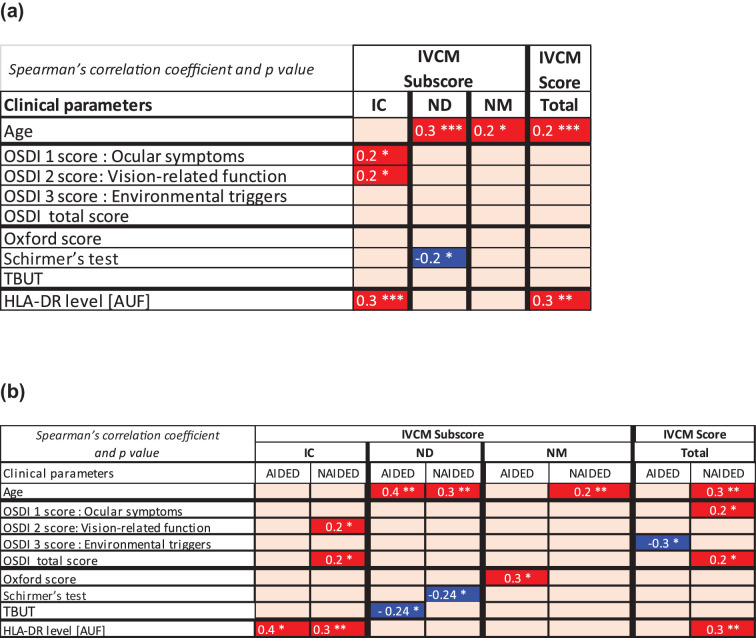
Correlations between IVCM scores and individual clinical indicators as well as with conjunctival HLA-DR expression in patients with DED. Significant correlations between parameters are represented by a correlogram for all patients, combining AIDED and NAIDED (**a**), and by separating DED subgroups according to their autoimmune status in AIDED and NAIDED (**b**). Statistically significant Spearman's correlation coefficients (*r*) are *highlighted in red* for a positive correlation and in *blue* for a negative correlation, whereas the empty boxes represent nonsignificant correlations. The *P* values are indicated as follows: *P < 0.05; **P < 0.01, ***P < 0.001. HLA-DR, human leukocyte antigen-DR; IC, inflammatory cells; IVCM, in vivo confocal microscopy; ND, nerve density; NM, nerve morphology; OSDI, Ocular Surface Index; TBUT, tear break up-time.

The clinical parameters and levels of HLA-DR were correlated with the T-IVCM score and its related subscores, ICsc, NDsc, and NMsc. Significant correlations are described in Figure 2a, by Spearman’s rank correlation coefficient (*r*). The T-IVCM score and ICsc showed significant correlation with HLA-DR levels, *r* = 0.3, *P* < 0.01, respectively. However, the T-IVCM score showed no correlation with either symptoms or signs. Nevertheless, the IVCM subscores showed significant correlation with signs and symptoms. Neuronal abnormalities, represented by nerve density (ND), were negatively correlated with Schirmer's test (*r* = −0.2, *P* < 0.05). ICsc, reflecting the inflammatory state, was positively correlated with both ocular symptoms and vision-related function OSDI subscores, both *r* = 0.2 and *P* < 0.05. Finally, patient age was correlated with nerve abnormalities through its correlation with NDsc and NMsc as well as the T-IVCM score (*r* = 0.23, *P* < 0.001). However, no correlation was found between inflammatory state, represented by ICsc, and age. This proposed grading system also shows a close result when a linear quantification grade was performed (Supplementary Tables S1a and S1b). The correlation for HLA DR level and density of ICs/mm² remain significant *r* = 0.2 and *P* < 0.05 as well as nerve length with the Schirmer strip *r* = 0.2 and *P* < 0.05 and aging *r* = −0.2 and *P* < 0.01.

### Comparisons Between Patients With AIDED and Patients With NAIDED Through Correlations Between IVCM Total Score and Subscores

Correlations between T-IVCM scores and clinical parameters were further analyzed by considering patients with DED according to their auto-immune status. Significant correlations are reported in Table 2b. ICsc showed the most significant correlation with HLA-DR levels in both groups (*r* = 0.4, *P* < 0.05) in patients with AIDED and (*r* = 0.3, *P* < 0.01) in patients with NAIDED. Nevertheless, the correlation between T-IVCM scores and HLA-DR levels was significant only in patients with NAIDED (*r* = 0.32, *P* < 0.05).NDsc was preferentially and negatively correlated with TBUT in patients with AIDED (*r* = −0.2, *P* < 0.05) and with Schirmer's test in patients with NAIDED (*r* = −0.24, *P* < 0.05). NMsc was positively correlated only with Oxford score in patients with AIDED (*r* = 0.3, *P* < 0.05). Finally, the correlation between T-IVCM score and subscores with OSDI showed a different distribution between groups. The T-IVCM score was correlated with the OSDI (*r* = 0.2, *P* < 0.05), exclusively with ocular symptoms of patients with NAIDED (*r* = 0.2, *P* < 0.05). Nevertheless, a negative correlation was seen with the OSDI environmental triggers score (*r* = −0.3, *P* < 0.05) only in patients with AIDED. Likewise, ICsc was correlated exclusively in patients with NAIDED.

### Conjunctival Expression of HLA-DR Within IVCM Subscore Groups

**Figure 3.**
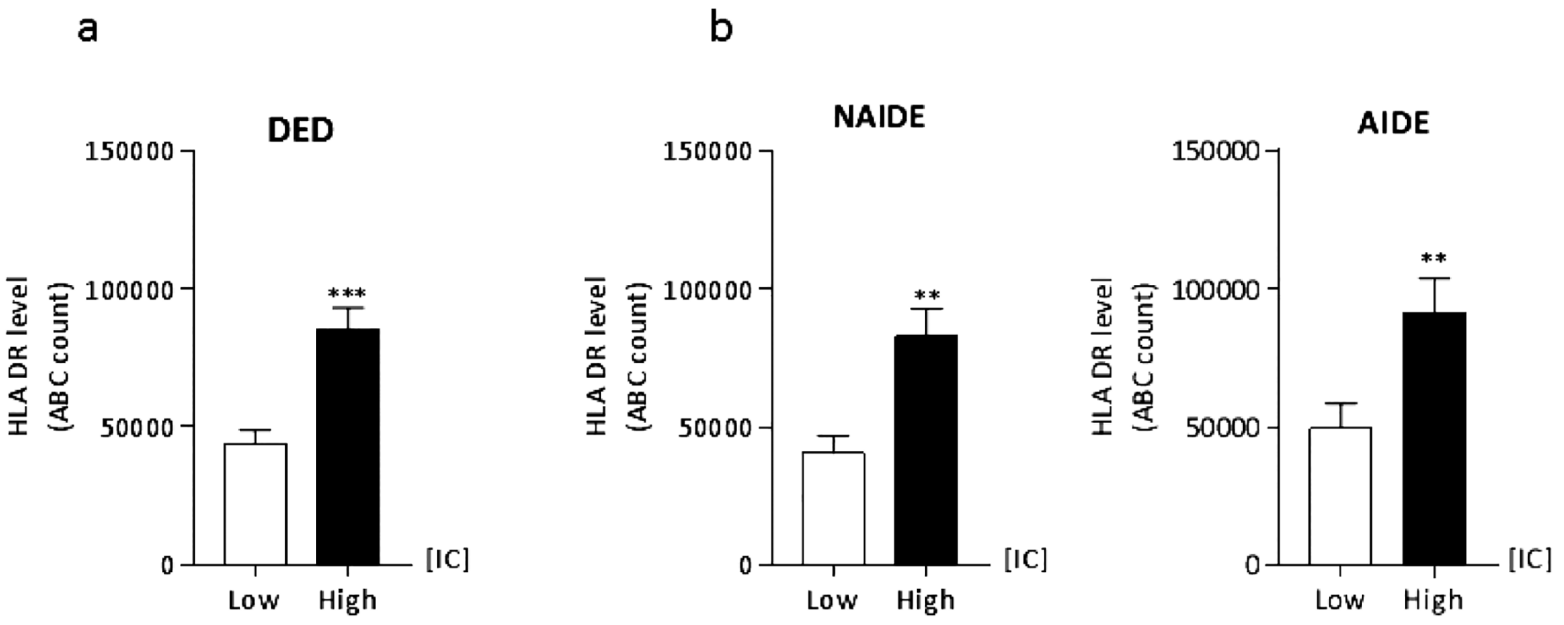
Conjunctival expression of HLA-DR according to IVCM-ICsc, which is related to ICs. Comparison between low IVCM-ICsc (IC-0 to IC-2) and high IVCM-ICsc (IC-3 to IC-4) showed a significant increase in conjunctival expression of HLA-DR in all patients with DED (**a**), as well as in both subgroups (**b**). Error bars represent standard errors of the mean (SEM). Data are expressed as mean ± SEM, and nonparametric comparisons between groups using the Mann-Whitney test were performed, with significant *P* values as follows: *P < 0.05; **P < 0.01; ***P < 0.001. AIDED, autoimmune dry eye disease; DED, dry eye disease; NAIDED, non-autoimmune dry eye disease.

To better characterize the association between corneal and conjunctival changes, the morphometric criteria presented in Figure 1 and Table 2 were considered. Similarly, after analyzing the global correlation between IVCM score and subscores and the clinical and molecular parameters, a specific stratification according to IVCM subscores, ICsc, NDsc, and NMsc, was performed. The resultant comparison between low ICsc (IC-0 to IC-2) and high ICsc (IC-3 to IC-4) showed a significant increase in conjunctival expression of HLA-DR in all patients with DED (Fig. 3a) as well as in both subgroups (Fig. 3b). Nevertheless, no significant difference was observed when stratification of the patients with DED was performed using corneal sensory nerve IVCM subscores (data not shown).

### Correlations Between Clinical Signs and Symptoms Among the Highest T-IVCM Scores

**Figure 4.**
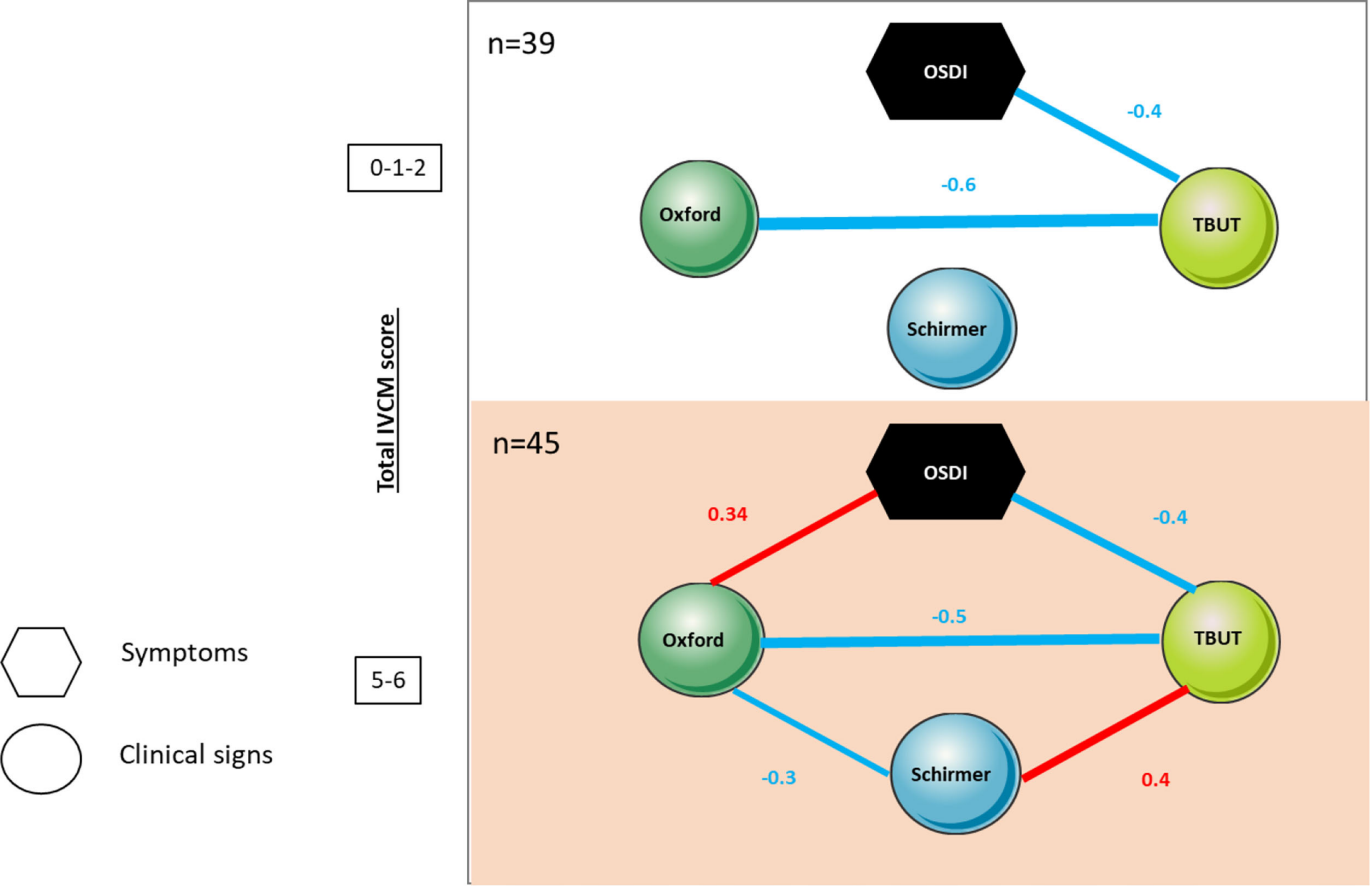
Summary of correlations between IVCM score and clinical parameters among low and high IVCM scores. Statistically significant correlations between clinical parameters are presented, including clinical symptoms and signs (OSDI, Oxford score, Schirmer test, and TBUT). The significant Spearman's correlation factor r is indicated between each set of parameters. Positive correlation is represented by *red lines*, and negative correlation by *blue lines*. Correlation strength is proportional to the thickness of the lines.

In order to enrich the meaningfulness of the IVCM scores, the patients with DED were stratified according to their total scores. Moreover, in order to homogenize the composition of the subgroups, extreme values of the T-IVCM score were considered separately, allowing the formation of two groups: group 1, consisting of the 39 eyes with the lowest T-IVCM scores (≤2), and group 2, consisting of the 45 eyes with the highest T-IVCM scores (≥5). Figure 4 summarizes the significant correlations between symptoms and signs in these two groups stratified according to their total IVCM scores. In both groups of patients with DED, TBUT values show a similar negative correlation between OSDI scores and Oxford test results. Nevertheless, in group 2, a new significant correlation appeared between OSDI and Oxford test (*r* = 0.34, *P* < 0.05). Additionally, the Schirmer's test was positively correlated with TBUT (*r* = 0.4, *P* < 0.01) and negatively correlated with the Oxford test (*r* = −0.34, *P* < 0.05) and TBUT (*r* = 0.4, *P* < 0.01). These results highlight the importance of IVCM morphological criteria in severe DED, taking into account the increase in DCs and the alterations in corneal sensory nerves.

## Discussion

In this study, we aimed to develop and evaluate a new IVCM scoring system based on nerve abnormalities and inflammatory cell density. Indeed, in recent years, growing interest in nerve damage and inflammation have encouraged the search for new biomarkers in DED.^23^^,^^25^^,^^43^^,^^44^ Thus, in order to develop a simplified imaging metric analysis, the proposed IVCM score took into consideration the densities of both ICs and sensory nerves as well as morphological nerve damage, to reflect corneal changes in DED. Thus, the T-IVCM score was subdivided into three subscores as follows: (i) ICsc, (ii) NDsc, and (iii) NMsc. The T-IVCM, as well as its related subscores, were subjected to correlation analyses with both clinical patterns and conjunctival HLA-DR levels in patients with DED.

We demonstrated a significant correlation between some clinical parameters and the molecular assessment with the T-IVCM score and subscores. The significant correlation between IVCM scores and clinical parameters was consistent with previous studies of sub-basal nerves^25^ and corneal DCs.^45^ Indeed, patient age was correlated with decreased NDsc and alterations in nerve morphology, and an increase in DCs was positively correlated with OSDI scores and subscores. Likewise, HLA-DR levels were higher in the presence of higher numbers of DCs in the cornea, which confirms the involvement of adapted immune responses during DED progression. The close association between conjunctival HLA-DR expression and age in patients with DED confirms the physiological link between inflammation and disease severity, as well as the aging process.^46^^–^^50^ Interestingly, our results highlight the fact that DED symptoms, qualified by total OSDI score, are correlated with IVCM-ICsc, supporting the relationship between inflammation and subjective symptoms. Additionally, the use of IVCM-ICsc, related to DC density, separately appears to be more suitable for estimating corneal changes compared to the sensory nerve subscore, IVCM-NDsc, alone. Indeed, even if nerve alterations reflect a pathologic state, quantification of them remains subjective. A recent review^19^ compiling corneal IVCM studies, in which the authors used nerve alterations and inflammation as principal parameters to differentiate DED subtypes, has suggested that using DCs is a better indicator of DED associated with systemic immune-mediated processes. Indeed, infiltration of activated DCs within the cornea represents an important sign for defining corneal inflammation, DCs being key players in the regulation of cell-mediated immunity.^27^^–^^29^ Likewise, inflammatory cellular components are considered excellent indicators of inflammatory activity and clinical severity.^16^^,^^50^ Interestingly, the ratio of immature to mature DCs, defined by their cellular shape, has been used to stratify patients with infectious keratitis.^51^ Additionally, the positive significant correlation between T-IVCM score, and specifically ICsc, with conjunctival HLA-DR expression reflects the close relationships between the mucosal response of the conjunctiva with the immune response in the cornea. Likewise, highly expressed HLA-DR in the conjunctiva was associated with an increase in corneal DCs under conditions of inflammation. This reflects the perpetuation of inflammation in the ocular surface. Similarly, decreased conjunctival HLA-DR expression after cyclosporine treatment was associated with ICsc decrease in the cornea (Supplementary Fig. S1). The close association between corneal changes and HLA-DR increase encourages and justifies further research on some promising targets of interest involved in inflammation, as previously identified by our team.^13^^,^^52^ Moreover, corneal sensory nerve parameters were preferentially related to DED signs, such as the quantity and quality of the tear film (TF). This observation reinforces the involvement of corneal nerves in TF homeostasis.^53^^–^^55^ Likewise, corneal innervation is vital for maintenance of corneal health and is involved in the protective mechanisms against harmful factors, as well as being essential for epithelial and endothelial integrity.^56^^–^^58^ Neurosensory dysfunction also contributes to DED.^1^ Nevertheless, across the literature, none of these sub-basal nerve metrics have been consistent in differentiating specific DED subtypes.^19^ This lack of specificity is due to both the heterogeneity within each etiology and the overlapping of clinical features between them. More discriminative criteria are needed to elucidate the significance of these corneal sensory nerve alterations.

In the second part of our study, patients with DED were clustered into two groups, patients with AIDED and patients with NAIDED, and similar relationships were confirmed. Interestingly, the positive correlation between the Oxford score and NMsc appeared to be more obvious in the AIDED group. These results reinforce the interest in deciphering the close connection between altered corneal epithelial cells and sensory nerve morphometric changes in chronic DED.^34^^,^^59^^,^^60^ Interestingly, NDsc was more closely related to TF volume in patients with NAIDED and to TF quality in patients with AIDED. However, the IVCM score and its subscores do not help in distinguishing between systemic auto-immune and non-autoimmune status. These observations have also been reported in several studies based on these corneal parameters in patients with DED.^44^ Moreover, the use of IVCM subscores was helpful in distinguishing between two main groups of patients showing sensory nerve alterations with or without inflammation. Interestingly, conjunctival HLA-DR expression was significantly different between these two groups. Nevertheless, no significant differences were reported in terms of clinical signs and symptoms between both groups (data not shown). However, the correlations between signs and symptoms within each group were significantly different. Indeed, the highest T-IVCM scores were related to a more severe state with an exacerbation of all ocular surface criteria. These observations highlight the fact that both alterations in nerves and increases in ICs in the cornea are associated with more severe DED. These results support the use of a simplified IVCM score to evaluate disease severity. Moreover, consideration of IVCM subscores separately might be helpful in understanding the relationship between associated symptomatology and morphological changes as the disease progresses. This also argues in favor of early evaluation of the cornea to assess the onset of abnormalities, whether in the presence or absence of inflammation.

Among the key points of our study, it is worth mentioning the value of evaluating this IVCM score in a longitudinal study or clinical trials to appreciate slight morphometric changes in regard to signs and symptoms. Similarly, it might be interesting to pursue the description of this score as a monitoring tool for evaluating topical or systemic treatments or other etiologies of DED, such as environmental exposures^61^ or contact lens wear.^62^ Nonetheless, the heterogeneity in terms of disease chronicity and the panel of treatments and duration of the included population could represent a limitation of the study. Moreover, the field of molecular biomarker research of the ocular surface will be strengthened with the additional clinical phenotyping available through this IVCM score. Indeed, the correlations between emerging molecular targets of interest and morphometric alterations in the cornea will shed light on pathophysiological mechanisms involved in the initiation and perpetuation of the disease. Interestingly, for future exploration, there may be a role for machine learning, which has been shown to be effective in classification by evaluating corneal nerves and DCs.^63^^,^^64^ This stratification will be used to analyze a high number of images and bring a modern approach to DED through artificial intelligence.

## Conclusions

In this study, we propose the use of a simplified IVCM score and subscore system to quantify morphometric alterations in the corneas of patients with DED. The clinical relevance of this simplified IVCM score was evaluated by the analysis of its correlation with signs and symptoms as well as with the inflammatory response as measured by conjunctival HLA-DR expression. Our findings suggest that the use of this IVCM score, particularly the consideration of its separate subscores, may be a useful method to establish a stratification system for DED diagnosis and biomarker investigation.

## Supplementary Material

Supplement 1

Supplement 2
