# Supplementary material for: Multimodal Approach in Dry Eye Disease Combining In Vivo Confocal Microscopy and HLA-DR Expression
Source: Transl Vis Sci Technol. 2024 Aug 23;13(8):39. doi: 10.1167/tvst.13.8.39 (PMC11346170; doi:10.1167/tvst.13.8.39)

Figure S1. Conjunctival expression of HLA-DR in patient with or without Cyclosporine treatments. Data are expressed as mean ± SEM, and non-parametric comparisons between groups using the Mann-Whitney test were performed, with significant p-values as follows:
*$p < 0.05$

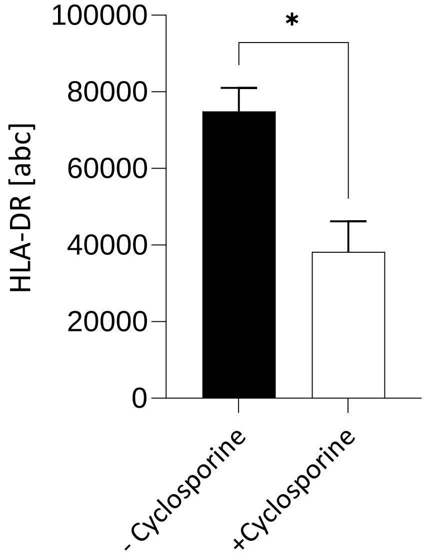

Supplement: Supplement 1 [file tvst-13-8-39_s001.pdf]
