# Supplementary material for: Multimodal Approach in Dry Eye Disease Combining In Vivo Confocal Microscopy and HLA-DR Expression
Source: Transl Vis Sci Technol. 2024 Aug 23;13(8):39. doi: 10.1167/tvst.13.8.39 (PMC11346170; doi:10.1167/tvst.13.8.39)
Supplement: Supplement 2 [file tvst-13-8-39_s002.pdf]

**Table S1.a**

| | *Spearman's correlation coefficient and p-value* | | |
|---|---|---|---|
| **Clinical parameters** | **NFD** (number of nerves/mm²) | **NFL** (mm/mm²) | **Inf. Cells (c/mm²)** |
| Age | **-0.22** ** (*0.004*) | **-0.22** ** (*0.004*) | -0.02 (*0.82*) |
| OSDI-1 score Ocular symptoms | -0.03 (*0.696*) | -0.004 (*0.960*) | 0.12 (*0.138*) |
| OSDI-2 score Vision-Related function | 0.01 (*0.846*) | 0.07 (*0.363*) | 0.14 (*0.06*) |
| OSDI-3 score Environmental Triggers | 0.07 (*0.397*) | 0.1296 (*0.100*) | -0.04 (*0.637*) |
| OSDI total | 0.003 (*0.964*) | 0.05 (*0.497*) | 0.12 (*0.121*) |
| Oxford score | -0.06 (*0.457*) | -0.10 (*0.202*) | **0.2** * (*0.02*) |
| Schirmer's test | 0.15 (*0.059*) | **0.2** * (*0.015*) | -0.06 (*0.457*) |
| TBUT | 0.15 (*0.050*) | 0.12 (*0.127*) | 0.05 (*0.547*) |
| HLA-DR level [AUF] | -0.07 (*0.397*) | -0.08 (*0.395*) | **0.2** * (*0.03*) |

*Abbreviation : Nerve fibre density (NFD) Nerve fibre length (NFL) ; Inf. Cells, c/mm² inflammatory cell count*

**Table S1.b**

| | AIDE | | | NAIDE | | |
|---|---|---|---|---|---|---|
| | ***Spearman's correlation coefficient and p-value*** | | | | | |
| **Clinical parameters** | **NFD** (number of nerve/mm²) | **NFL** (mm/mm²) | **Inf. Cells (c/mm²)** | **NFD** (number of nerves/mm²) | **NFL** (mm/mm²) | **Inf. Cells (c/mm²)** |
| Age | **-0.27*** (*0.05*) | **-0.25** (*0.075*) | 0.004 (*0.976*) | **-0.20*** (*0.029*) | **-0.22*** (*0.017*) | -0.01 (*0.870*) |
| OSDI 1 score Ocular symptoms | -0.08 (*0.544*) | 0.03 (*0.791*) | 0.12 (*0.382*) | 0.01 (*0.903*) | -0.005 (*0.950*) | 0.13 (*0.149*) |
| OSDI 2 score Vision-Related function | -0.09 (*0.487*) | -0.05 (*0.679*) | -0.07 (*0.608)* | 0.09 (*0.311*) | 0.13 (*0.147*) | 0.23 (*0.013*) |
| OSDI 3 score Environmental Triggers | 0.18 (*0,202*) | **0.35** * (*0,01*) | -0.17 (*0,232*) | 0.05 (*0,604*) | 0.09 (*0,32*) | 0.03 (*0,768*) |
| OSDI total | -0.04 (*0.765*) | 0.12 (*0.425*) | -0.03 (*0.809*) | 0.05 (0.630) | 0.06 (*0.55*) | **0.21*** (0.028) |
| Oxford score | -0.06 (*0.658*) | -0.06 (*0.687*) | 0.19 (*0.159*) | -0.001 (*0.992*) | -0.084 (*0.379*) | **0.23*** (*0.01*) |
| Schirmer's test | -0.08 (*0.56*) | 0.01 (*0.96*) | -0.23 (*0.100*) | **0.17** (*0.06*) | **0.22*** (*0.02*) | -0.04 (*0.666*) |
| TBUT | 0.15 (*0.26*) | 0.14 (*0.32*) | -0.21 (*0.136*) | 0.14 (*0.126*) | 0.13 (*0.15*) | 0.14 (*0.143*) |
| HLA-DR level [AUF] | 0.29 (*0.11*) | **0.35** (*0.06*) | **0.41** * (*0.02*) | **-0.17** (*0.07*) | **-0.12**** (*0.01*) | 0.11 (*0.266*) |

*Abbreviation : Nerve fibre density (NFD) Nerve fibre length (NFL); Inf. Cells, c/mm² inflammatory cell count*
